# Adaptive capacity of Asian populations of *Lymantria dispar* to non preferred plants during northward expansion

**DOI:** 10.1038/s41598-025-32504-x

**Published:** 2026-01-16

**Authors:** E. L. Arzhanova, S. V. Pavlushin, I. A. Belousova, Y. B. Akhanaev, A. S. Bastrygina, V. V. Martemyanov

**Affiliations:** 1 Research Center for Genetics and Life Sciences, Sirius University of Science and Technology, Olympic Prospect 1, Sirius Federal Territory, Sochi, Russia 354340; 2 Institute of Systematics and Ecology of Animals SB RAS, Frunze Str. 11, Novosibirsk, Russia 630091; 3 Novosibirsk State University, Pirogova 2, Novosibirsk, Russia 630090

**Keywords:** Climate change, Fitness traits, Host plant adaptation, *Lymantria dispar*, Physiological plasticity, Range expansion, Ecology, Ecology, Plant sciences

## Abstract

The spongy moth (*Lymantria dispar*) is one of the most widespread insect defoliators in the Holarctic, with a capacity to form regular large-scale outbreaks. In the context of global climate change, the geographic range of this species is undergoing a shift that necessitates adaptation to novel host plants, including conifers. The present study assessed the capacity of insects from two *L. dispar* populations to use pine and larch as hosts, namely *Pinus sylvestris* and *Larix sibirica*, as compared to their habitual birch host, *Betula pendula*. The survival rate of larvae from a Novosibirsk population (south Siberian steppe) was similar on birch and larch, but lower on pine. In contrast, the survival of larvae from an Altai population (south Siberian mountainous landscape) was similar on all three hosts. However, females from both populations exhibited a reduced tendency to oviposit on pine compared to birch and larch, with no discernible differences observed between larch and birch. Hatching success was found to be independent of the host plant. Feeding on pine significantly reduced pupal mass and prolonged larval development compared to birch and larch in both populations. Physiological correlates of adaptation to new host plants were also examined. In 4th instar larvae, alkaline protease activity increased on larch and pine compared to birch but decreased on pine relative to larch, showing no population-specific tendency. Esterase activity exhibited variation exclusively in males, while malondialdehyde accumulation increased for both populations on larch. The findings reported here show that the spongy moth exhibits sufficient physiological adaptability to utilize coniferous hosts, thus highlighting its potential to expand into new ecological niches under changing environmental conditions.

**Supplementary Information:**

The online version contains supplementary material available at 10.1038/s41598-025-32504-x.

## Introduction

Climate change has been documented for a considerable period^1,2^, with global-average surface temperatures increasing by approximately 0.6 °C over the past century^2^. In recent decades, a growing body of research has demonstrated the impact of global warming on insects^3–6^. Bark beetles and moths represent just a partial list of pests that have caused extensive damage to deciduous and coniferous forests across millions of hectares in North America and Fennoscandia over the past 20 years^7–10^. The primary cause of these outbreaks is the positive effect of climate warming on key stages of insect life cycles, facilitating unprecedented large-scale and hazardous population outbreaks. A of the most severe consequences of global warming is the transitioning of boreal forests to an alternative ecosystem state^11–13^. For instance, there has been a shift to a treeless secondary tundra, as was observed in northern Finland during outbreaks of the autumnal moth, *Epirrita autumnata*, in the 1960s^14^.

One of the most prevalent phytophagous insects in the Holarctic region is the spongy moth (previously known as the gypsy moth) *Lymantria dispar*^15^, which periodically forms outbreak areas across hundreds of thousands of hectares^16,17^. Projections derived from CLIMEX modelling suggest the possibility of the spongy moth extending its range by hundreds of kilometers north under a scenario of a 2–3 °C rise in average annual temperature^18^. Subsequent studies utilizing pheromone monitoring techniques to observe the northern boundary of the Asian *L. dispar* population have corroborated the documented northward shift in the species’ geographical distribution^19,20^. The mean rate of this northward expansion is estimated to be approximately 50 km per year^20^.

It is well established that the spongy moth is a highly polyphagous species, with a capacity to use hundreds of plant species as hosts^15^. However, despite its broad polyphagy, populations of the spongy moth exhibit clear preferences for dominant woody plants in different parts of its range. For instance, in Western Siberia, the spongy moth attacks primarily damage silver birch (*Betula pendula* Roth.). However it is known that during outbreaks spongy moths also consume coniferous foliage, and these trees tend to be much less tolerant to defoliation^21^. As the vegetation transitions northwards from the boreal to the light coniferous taiga zone, we observe a decrease in the proportion of birch and an increase in the proportion of conifers. Although the boundaries of the taiga are also expected to shift northward over time^22^, the rate of these changes, in slow-growing trees with multi-year developmental cycles, will be significantly lower than that of insects. Additionally, expanding insect populations will encounter new species of host plants whose leaf chemistry might depend on the latitude under which they grow^23^. However, our recent studies show that most important phytochemical toxicants will not be a serious barrier to the northward expansion of spongy moths normally feed on *B. pendula* and *Larix sibirica* Ledeb. populations^24^. The adaptation of the spongy moth to feeding on conifers is of particular significance, as coniferous trees generally exhibit reduced tolerance to total defoliation in comparison to deciduous species, resulting in widespread dieback across extensive regions (referred to as “silkworm squares”)^25^. Furthermore, the relatively high frequency of spongy moth outbreaks^16,26,27^ could potentially result in a catastrophe for boreal forests should this species successfully invade and adapt to new environments.

It has been demonstrated that the spongy moth exhibits significant ecological plasticity, as evidenced by its ability to adapt to North American^28,29^, and certain European conifer species^30^. However, recent studies indicate that Asian northern populations respond more favorably to regional thermal conditions compared to North American populations^20^. Specifically, males from Asian populations exhibit minimal shifts in flight timing, whereas male moths from North American populations exhibit a pronounced shift in their flight period towards autumn as they migrate northwards^20^. These differences may be associated with a lowered temperature threshold for late embryonic development in Asian *L. dispar* populations^31^.

The activity of enzymes involved in food digestion and detoxification of xenobiotics can be used to assess the degree of adaptation to a new host plant. Feeding on unsuitable plants may necessitate the synthesis of proteases isozymes^32^ or higher levels of proteases for efficient protein breakdown^33^ and to counteract protease inhibitors^34^, albeit at the cost of increased energy expenditure. It has been demonstrated that the protein and carbohydrate contents^35^ of a diet and plant secondary metabolites^36,37^ has a significant impact on the activity of proteases. Furthermore, the intake of alien secondary plant metabolites requires detoxification, which is facilitated by esterases that catalyze the hydrolysis of ester bonds in xenobiotics, thereby increasing their water solubility and preparing them for subsequent conjugation. Gut pH plays a critical role in digestive efficiency in insect herbivores, influencing enzyme activity, nutrient solubilization, and the stability of ingested secondary metabolites^38^. The ingestion of plant material introduces both pro-oxidant and antioxidant compounds into the insect gut, and the balance between these components can modulate redox conditions and, consequently, luminal pH. Changes in gut pH, in turn, may affect the activity of digestive enzymes and the bioavailability of nutrients, thereby shaping overall digestive performance and physiological responses in herbivorous insects. This, in turn, has been shown to have both direct and indirect impacts on an insect’s capacity to adapt to a novel host plant.

The objective of the present study was to evaluate the ability of the spongy moth to switch from its common host, silver birch, to two coniferous species, the Siberian larch and Scots pine, and to assess the persistence of any observed adaptive response in the next generation. In addition, given that some Siberian populations of the spongy moth are able to use larch as a host, we compared one such population with one that typically feeds exclusively on broad-leave trees. Finally, in order to determine whether physiological mechanisms are triggered by such host switches, we compared two populations with respect to the activity of some digestive and detoxification enzymes and the level of oxidative stress in the insect gut when feeding on different plant species.

## Materials and methods

### Insects

**Fig. 1:**
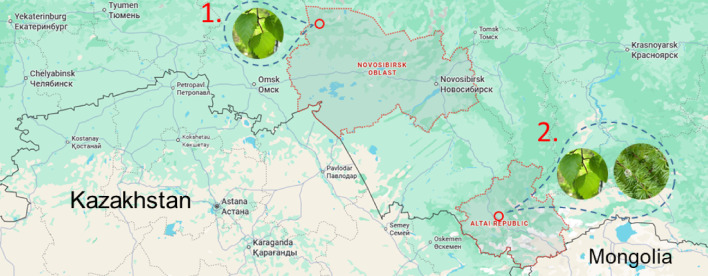
Geography and general locations of the populations and host plants. **1**. Kyshtovka (Novosibirsk Oblast): Novosibirsk “flat” population, *Betula pendula* Roth.—preferable host plant. **2.** Ongudai (Altai Republic): Altai “mountain” population, *Betula pendula* Roth. and *Larix sibirica* Ledeb.—are preferable host plants (Map data ©2025 Google).

The spongy moth (*Lymantria dispar*) is neither a protected nor a rare species, according to the Red Data Book of Russia and the International Union for Conservation of Nature (IUCN). The European Convention for the Protection of Vertebrate Animals Used for Experimental Purposes (Directive 2010/63/EU) does not regulate the use of insects in research; however, all work was conducted in accordance with internal laboratory standards that aim to minimize animal suffering during experiments. Egg masses were collected in natural conditions during the autumn and stored in the laboratory at 0 to + 4 °C. For this study, we selected two populations of the spongy moth. The first population (Novosibirsk “flat” population) was collected in a lowland area near the northern border of the Novosibirsk region (N 55° 40′ 52.6584" E 76° 45′ 6.0444") (Fig. 1), effectively at the northern boundary where the species is capable of forming mass outbreaks^16^. If northward expansion continues, this population will face the necessity of switching its host plant to coniferous species. Its currently preferred host plant is silver birch. The second population (Altai “mountain” population) was collected from mountainous regions of the Altai (N 50°45′00″ E 86°09′00″) (Fig. 1). The Altai population was chosen as a model of a more ecologically plastic population, as outbreaks of the spongy moth in this mountainous area have been observed on both silver birch and Siberian larch. Recent population genetic studies have shown that the Altai population forms a distinct genetic cluster^39^. Although these two populations outwinter under different environmental conditions, the temperature regimes during hibernation do not differ significantly. Specifically, Siberian populations overwinter at the base of tree trunks under a layer of snow and their adult females are flightless, while Altai populations overwinter on rocky outcrops^40^ and their adult females are flight capable. In the first case, the population is protected from extreme low temperatures by the insulating snow layer^41^, while in the second case, the buffering capacity of rocky outcrops provides protection^40^.

### Host plants and experimental design

The collection of plant material was conducted in accordance with local legislation pertaining to biodiversity and nature conservation. A survey of the plant species utilized in the study reveals that none of them are classified as endangered or rare according to the Red Data Book of Russian Federation or the IUCN. The species affiliation of plants was determined by Dr. Martemyanov according to morphological features. Consequently, deciduous forests within the forest-steppe zone are predominantly composed of silver birch, with no overlap observed in the distribution of other species belonging to *Betula* spp.^42^. The species of pine that are characteristic of this zone, *Pinus sibirica* and *Pinus sylvestris* (Scots pine), exhibit striking visual differences in needle length. *Larix sibirica* is the sole larch species in this region^43^. Silver birch was selected as the control host plant, being the primary host consumed by both the Novosibirsk and Altai populations of the *L. dispar*. Scots pine was used as a model of a potential host plant in the conditions of the West Siberian light-coniferous taiga. Siberian larch was chosen as an “intermediate” option, as it is already known to be damaged by the spongy moth in some regions, including the Altai region.

In May, the insects were brought out of diapause. To avoid developmental delay on non preferred host plants prior to the experiments and to reduce high mortality in the first larval instar, the insects were reared on young birch leaves until they reached the second and third instars. No significant differences were observed in the experimental data between groups fed on pine from second and third instars, so the groups were combined. In the second instar, the insects were placed into plastic containers (5 L in volume) with 20 individuals per container. Each population was divided into three feeding groups based on diet: birch, larch, and pine. Foliage was changed on average every 3–4 days. Inside the container, plant stems were inserted into 50 ml Falcon centrifuge tubes, and the tubes were tightly wrapped with Parafilm to prevent water spillage. The temperature was maintained at + 21–22 °C, and the humidity was 60%.

A total of 340 insects from each population were reared to assess viability until pupation. Variables such as pupal mass one day after pupation, larval stage duration, survival rate, individual fecundity, and offspring survival were evaluated. Additionally, a separate group of insects from each population was reared on the same three host species to study variables upon reaching the fourth larval instar.

After adult emergence, moths were arranged in mating pairs to obtain individual egg clutches. After the summer embryonic maturation, the eggs were overwintered at 0 °C in the “zero-degree zone” of the standard refrigerator and, in the spring of the following year, the second-generation egg masses were brought out of diapause. Individual fecundity (number of eggs per clutch) and hatching success of both generations were recorded. A total of 30 egg clutches from each population were analyzed (10 clutches from each dietary group). Survival rate was assessed using five biological replicates per experimental group, with each replicate consisting of 20 insects.

Subsequently, the second generation was reared following the same protocol as the first generation until the third generation was obtained. The viability variables of the second generation were compared with those of the first generation. Physiological variables were not assessed in the second generation.

### Enzymes activity analysis

To assess physiological variables, 300 fourth-instar larvae from each population were selected (100 from each dietary group) and sacrificed one day after molting. As indicators of physiological adaptation to the change in host plant, we measured the activity of digestive enzymes (alkaline proteases), detoxification enzymes (esterases), and lipid peroxidation activity by quantification of malondialdehyde (MDA) accumulation. For the enzyme assays, larval midguts from each group were individually dissected in ice-cold potassium phosphate buffer (0.2 mol/L, pH 7.8, with 1 mmol/L EDTA), ground in an ultrasound tissue homogenizer (Bandelin SONOPULS HD 2070) and centrifuged for 10 min at 20,000 × g 4 °C. Each homogenate was prepared from a single midgut dissected from one individual insect. The sex of the larvae was determined by examining the gonads during midgut dissection for physiological parameters.

Alkaline protease activity was evaluated using a colorimetric method with azocasein (Sigma-Aldrich, USA, A2765) as the substrate^44^. The midgut homogenate supernatant was diluted sevenfold with buffer (0.1 M Tris–HCl, 150 mM NaCl, pH 8–9.6), followed by addition of 140 μl of azocasein solution in NaCl (20 mg/mL). The mixture was incubated for 25 min at 28 °C, after which the reaction was stopped by adding 40 μl of 30% trichloroacetic acid (Reachim, Russia) (TCA). The resulting solution was centrifuged for 10 min at 1000 × g, followed by the addition of 60 μl of 1 M NaOH to the supernatant. Absorbance was measured at 440 nm using microplate reader BioTek PowerWave HT (BioTek Instruments, USA).

The esterase activity in the midgut was measured using p-nitrophenyl acetate (Sigma-Aldrich, USA, N8130) as a substrate^45^. The reaction mixture (1 ml) consisted of 10 μl of supernatant of midgut homogenate and 200 μl of 0.01 M p-nitrophenyl acetate in 200 mM phosphate buffer (pH 7.2). The esterase activity was measured as the difference in absorbance at 410 nm using microplate reader after 30 min of incubation with the substrate at 28 °C.

Malondialdehyde (MDA) accumulation was also assessed using a colorimetric method with thiobarbituric acid (TBA) (Sigma-Aldrich, USA, T5500) as the substrate^46^. To 90 μl of midgut homogenate supernatant, 65 μl of 20% TCA was added, and the mixture was centrifuged for 2 min at 20,000 × g. From the supernatant, 120 μl was collected, and 80 μl of 0.8% TBA was added. The resulting solution was incubated for 20 min at 98 °C, and absorbance was measured at 532 nm using microplate reader.

### Statistical analysis

The data are presented as mean ± standard error (Mean ± SE) for normally distributed samples (number of eggs laid, pupal mass, and alkaline protease activity) and as box plots for data that do not fit a normal distribution (larval stage duration, esterase activity, and MDA accumulation), except for survival rate and hatching percentage, which are presented as Mean ± SE. Statistical analyses were performed using Statistica 12.0 (StatSoft, USA) and Past 4.10^47^ software. The Kolmogorov–Smirnov and Shapiro–Wilk tests were used to assess the normality of the distribution of quantitative data. For analyzing non-normally distributed samples, the Kruskal–Wallis test was applied, followed by Dunn’s test for post-hoc analysis. For normally distributed samples, Factorial ANOVA was used, with Fisher’s test for post-hoc analysis. Host tree, population and sex were set as categorical factors. Interaction of factors is stated only if significant. Survival rate (number of surviving and dead individuals per replicate) was analyzed using a generalized linear model with a beta-binomial distribution and logit link function. The model was fitted using the glmmTMB package^48^ in R^49^. Fixed effects included population, plant, and their interaction (population × plant) to assess both main and combined influences on insect survival. Significance of model terms was assessed using Wald chi-squared tests. Post-hoc pairwise comparisons for significant main effects were performed with Tukey-adjusted tests for multiple comparisons.

**Fig. 2:**
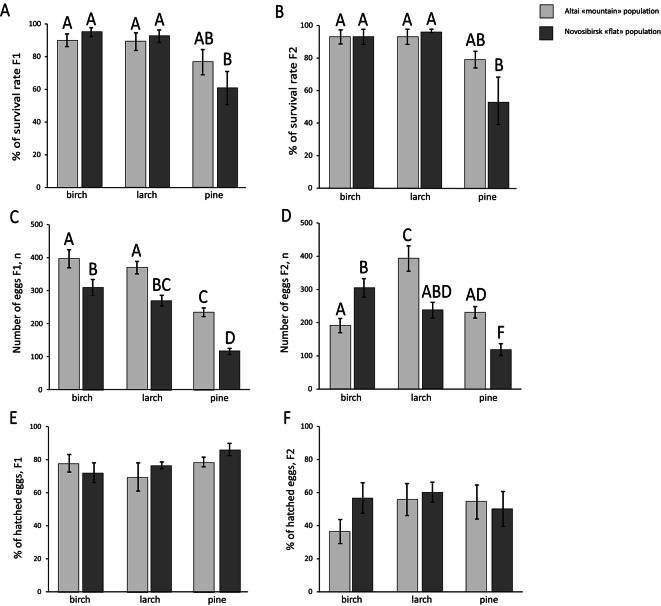
Population associated indices of adaptability to host plant changes in two generations of *Lymantria dispar*. (**A**) Survival rate of first-generation insects from two populations when fed on three host plants. (**B**) Survival rate of second-generation insects from two populations when fed on three host plants. (**C**) Number of eggs laid by first-generation insects from two populations when fed on three host plants. (**D**) Number of eggs laid by second-generation insects from two populations when fed on three host plants. (**E**) Hatching success of insects derived from eggs of the first generation of two populations when fed on three host plants. (**F**) Hatching success of insects derived from eggs of the second generation of two populations when fed on three host plants. Different letters indicate statistically significant differences; same letters indicate absence of statistically significant differences between the groups.

**Fig. 3:**
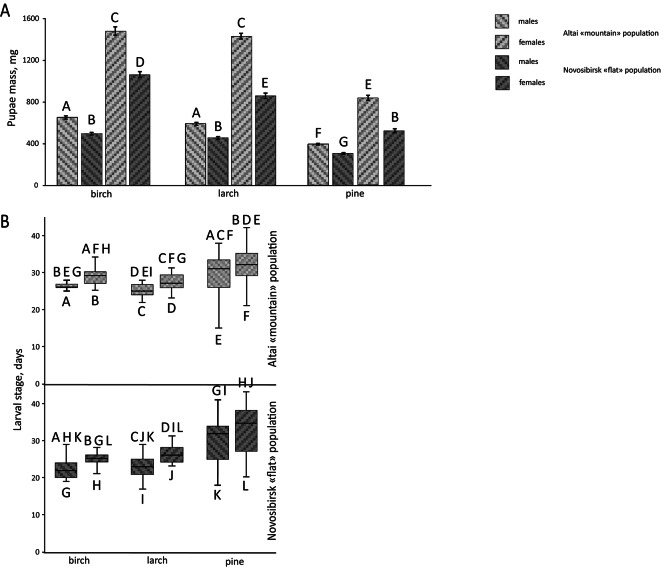
Life history traits of *Lymantria dispar* reared on different host plants. (**A**) Pupal mass of insects after feeding on three host plants. Different letters indicate statistically significant differences. (**B**) Larval stage duration of insects when fed on three host plants. Letters below the box plot are individual group identifiers, while letters above the box plot indicate groups that are statistically significantly different from this group.

**Fig. 4:**
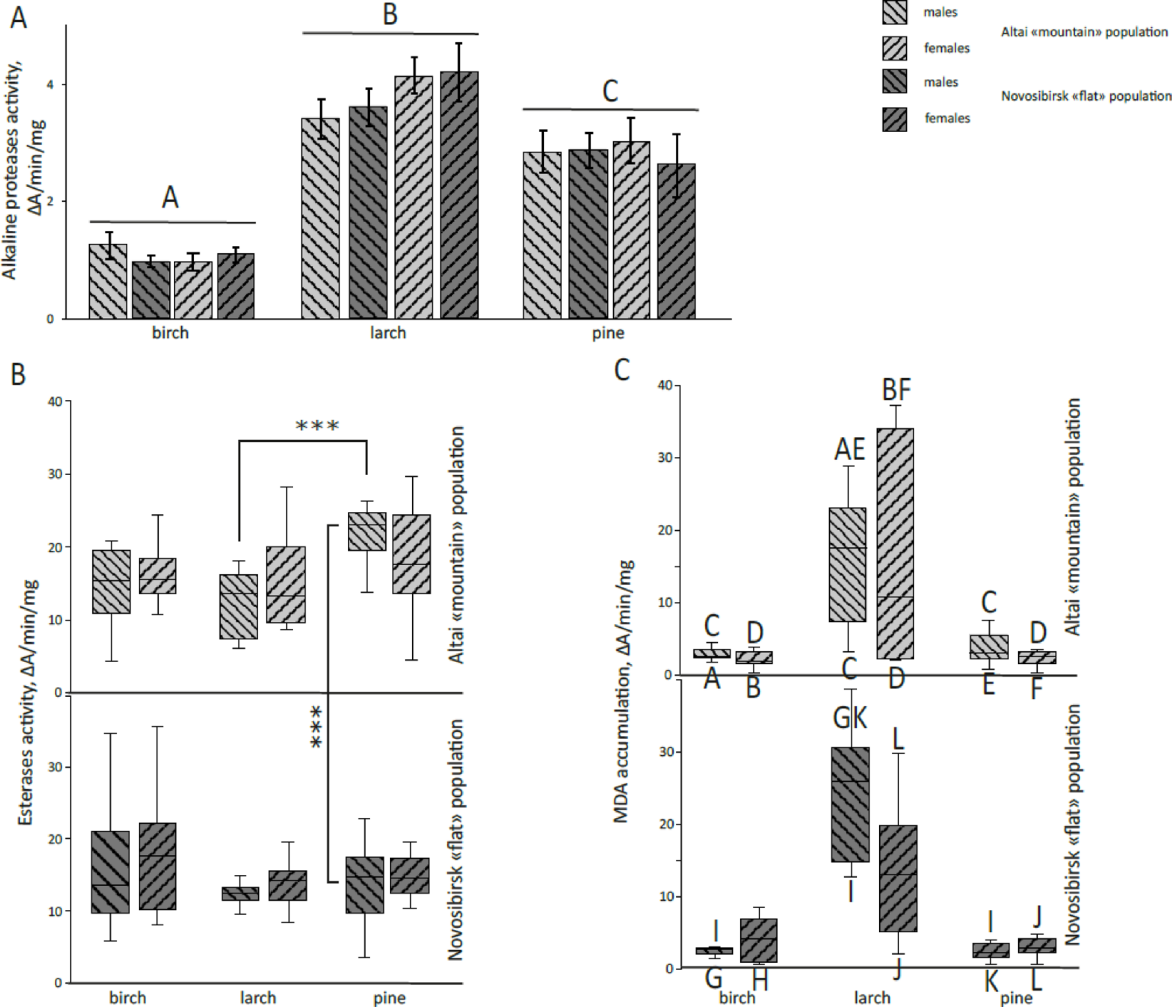
Physiological responses to feeding on different host plants in 4th instar *Lymantria dispar* female and male larvae originating from Altai and Novosibirsk populations. (**A**) Alkaline protease activity in the midgut when fed on different host plants. Different letters indicate statistically significant differences. (**B**) Esterase activity in the midgut when fed on different host plants. ***p < 0.001. (**C**) Malondialdehyde (MDA) accumulation in the midgut when fed on different plants. Letters below the box plot serve as individual group identifiers, while letters above the box plot indicate groups that are statistically significantly different from this group.

In Figs. 2A, B, C, D, 3A and 4A groups sharing the same letter are not significantly different (*p* > 0.05). In Figs. 3B and 4C each group is assigned a unique group identifier positioned below the corresponding box and above each group, the identifiers of the groups from which it differs significantly are indicated. Panels without letters indicate no significant differences among any groups.

## Results

### Fitness traits of studied populations

Survival and fecundity assessments were conducted on insects from two generations. In the first generation, survival was influenced solely by host plant species, as the Novosibirsk population exhibited markedly reduced survival on pine relative to birch and larch. (host tree effect W = 15.40, *df* = 2, *p* < 0.001, post-hoc Tukey’s test *p* < 0.001) (Fig. 2A). The survival rate of insects from both populations did not differ when fed on larch compared to birch (Fig. 2A). All trends observed in the first generation persisted in the second generation: survival of the Altai population on pine remained unchanged, while survival of the Novosibirsk population was, again, lower on pine than on birch and larch (population effect W = 4.06 *df* = 1, *p* < 0.05, host tree effect W = 23.18, *df* = 2, *p* < 0.001, post-hoc post-hoc Tukey’s test *p* < 0.01 ) (Fig. 2B).

The number of eggs laid by the first generation was lower on pine compared to birch and larch for both the Altai (Two-way ANOVA host tree effect F(2, 54) = 48.48 *p* < 0.001, post-hoc Fisher’s LSD test, *p* < 0.001) (Supplementary table 1) and Novosibirsk populations (Two-way ANOVA host tree effect F(2, 54) = 48.48 *p* < 0.001, post-hoc Fisher’s LSD test, *p* < 0.001) (Fig. 2C) (Supplementary table 1). However, the number of eggs laid on larch did not differ from that on birch for either population (Fig. 2C) (Supplementary table 1). Additionally, females of the Altai population laid more eggs than the Novosibirsk population across all three host plants (Two-way ANOVA population effect F(1, 54) = 42.22 *p* < 0.001, post-hoc Fisher’s LSD test, *p* < 0.01) (Fig. 2C) (Supplementary table 1). In the second generation, the Novosibirsk population laid fewer eggs on pine compared to birch and larch (Two-way ANOVA host tree effect F(2, 54) = 15.33 *p* < 0.001, factors interaction F(2, 54) = 16.20 *p* < 0.001, post-hoc Fisher’s LSD test, *p* < 0.01) (Supplementary table 2), but egg counts on larch and birch did not differ (Fig. 2D) (Supplementary table 2). For the second-generation Altai moths, the number of eggs laid did not differ between pine and birch, but was higher in larch compared to birch (Two-way ANOVA host tree effect F(2, 54) = 15.33, factors interaction F(2, 54) = 16.20 *p* < 0.001, post-hoc Fisher’s LSD test, *p* < 0.001) (Fig. 2D) (Supplementary table 2).

Hatching success, defined as the percentage of insects hatching from laid eggs, showed no differences between populations or host plants in either the first or the second generation (Fig. 2E, F).

Further studies were conducted on first-generation insects. Feeding on pine by the Altai population resulted in reduced pupal mass for both sexes (Factorial ANOVA host tree effect F(2, 599) = 415.00 *p* < 0.001, post-hoc Fisher’s LSD test, *p* < 0.01) (Supplementary table 3) compared to feeding on birch and larch (Fig. 3A) and increased larval stage duration for both sexes (Kruskal–Wallis test H = 287.81 *p* < 0.001, post-hoc Dunn’s test, *p* < 0.01 for males and Kruskal–Wallis test H = 287.81 *p* < 0.001, post-hoc Dunn’s test, *p* < 0.05 for females) (Fig. 3B). A similar effect was observed for the Novosibirsk population: feeding on pine reduced pupal mass for both males (Factorial ANOVA host tree effect F(2, 599) = 415.00 *p* < 0.001, post-hoc Fisher’s LSD test, *p* < 0.01) (Supplementary table 3) and females (Factorial ANOVA host tree effect F(2, 599) = 415.00 *p* < 0.001, post-hoc Fisher’s LSD test, *p* < 0.01) (Supplementary table 3) compared to feeding on birch and larch (Fig. 3A), and increased larval stage duration for both sexes (Kruskal–Wallis test H = 287.81 *p* < 0.001, post-hoc Dunn’s test, *p* < 0.01) (Fig. 3B).

Feeding on larch by Altai population did not reduce pupal mass for both sexes compared to feeding on birch (Fig. 3A) (Supplementary table 3). However, for the Novosibirsk population, female pupal mass decreased when fed on larch compared to birch (Factorial ANOVA host tree effect F(2, 599) = 415.00 *p* < 0.001, post-hoc Fisher’s LSD test, *p* < 0.01) (Supplementary table 3), while male pupal mass remained unchanged (Fig. 3A). Additionally, the larval stage duration for both sexes in the Altai population was longer compared to the Novosibirsk population when fed on larch (Kruskal–Wallis test H = 287.81 *p* < 0.001, post-hoc Dunn’s test, *p* < 0.01 for males and Kruskal–Wallis test H = 287.81 *p* < 0.001, post-hoc Dunn’s test, *p* < 0.05 for females) (Fig. 3B). However, the larval stage duration for both sexes in the Altai and Novosibirsk populations did not differ when fed on larch compared to birch (Fig. 3B).

### Physiological traits

The activity of alkaline proteases did not vary as a function of source population, but was strongly affected by the host plant, with activity being significantly higher in insects fed on larch and pine than in those fed on birch (Factorial ANOVA host tree effect F(2, 120) = 44.07 *p* < 0.001, post-hoc Fisher’s LSD test, *p* < 0.001) (Supplementary table 4), while significant differences were also observed between larch-fed and pine fed insects, with the former displaying higher activity (Factorial ANOVA host tree effect F(2, 120) = 44.07 *p* < 0.001, post-hoc Fisher’s LSD test, *p* < 0.01) (Fig. 4A) (Supplementary table 4).

Within the Altai population, although esterase activity was significantly higher in males fed on pine than in those fed on larch (Kruskal–Wallis test H = 25.00 *p* < 0.01, post-hoc Dunn’s test, *p* < 0.001), the host plant was found to have had no significant impact on activity in all other comparisons considered (Fig. 4B). Similarly, within the Novosibirsk population, esterase activity was not significantly different among insects of both sexes fed on pine and those fed on birch or larch (Fig. 4B). However, an inter-population comparison of esterase activity shows that Altai males fed on pine displayed higher activity than similarly fed Novosibirsk males (Kruskal–Wallis test H = 25.00 *p* < 0.01, post-hoc Dunn’s test, *p* < 0.001), while no differences are observed between females of the two populations (Fig. 4B).

MDA accumulation in the Altai population does not change when fed on pine compared to birch but decreases compared to feeding on larch for both sexes (Kruskal–Wallis test H = 51.08 *p* < 0.001, post-hoc Dunn’s test, *p* < 0.01 for males and Kruskal–Wallis test H = 51.08 *p* < 0.001, post-hoc Dunn’s test, *p* < 0.05 for females) (Fig. 4C). Additionally, MDA levels are higher in the Altai population when fed on larch compared to birch for both sexes (Kruskal–Wallis test H = 51.08 *p* < 0.001, post-hoc Dunn’s test, *p* < 0.01 for males and Kruskal–Wallis test H = 51.08 *p* < 0.001, post-hoc Dunn’s test, *p* < 0.05 for females) (Fig. 4C). In the Novosibirsk population, MDA accumulation does not change when fed on pine compared to birch but decreases compared to feeding on larch for both sexes (Kruskal–Wallis test H = 51.08 *p* < 0.001, post-hoc Dunn’s test, *p* < 0.001 for males and Kruskal–Wallis test H = 51.08 *p* < 0.001, post-hoc Dunn’s test, *p* < 0.05 for females). Additionally, MDA levels are higher when fed on larch compared to birch in males (Kruskal–Wallis test H = 51.08 *p* < 0.001, post-hoc Dunn’s test, *p* < 0.001) (Fig. 4C). No differences in MDA accumulation are observed between populations on any host plant.

## Discussion

The northward range expansion of the spongy moth (*Lymantria dispar*) and the potential shift in host plants have recently attracted much research attention. In particular, the ability of European and North American spongy moth populations to feed on conifers or other plant species containing specific secondary metabolites has been closely examined^28^. It has been demonstrated that certain secondary metabolites belonging to the class of monoterpenes are toxic to lepidopteran larvae. These metabolites have been shown to reduce feeding efficiency and to slow the growth and development of the larvae^50,51^. Moreover, the presence of mono- and diterpenes in larch has been shown to reduce the palatability of food, consequently leading to a decrease in weight gain in spongy moth larvae^52^. When larvae are fed on novel or unsuitable host plants, their digestive enzyme profiles change. Depending on the profile of plant secondary metabolites, the activity of both digestive and detoxification enzymes may be altered^53^.

According to an earlier report^54^, it takes approximately 40 generations (i.e., 40 years, as *L. dispar* is strictly univoltine) for European populations of spongy moth to develop a measurable degree of adaptation to a host plant previously outside their natural diet. Studies on the host shift of the spongy moth to non-preferred plant species have shown that, even after 40 generations and despite the development of partial physiological adaptations, insects still exhibit a strong preference for their original host plant^54^. Larch is regarded as a suboptimal host for the spongy moth, as the synthesis of digestive and detoxification enzymes demands additional energy, which would otherwise be directed towards biomass accumulation^55,56^. The nutrient efficiency of spongy moth larvae feeding on larch is not significantly different from that of larvae feeding on pine, but it is more than twice lower than that assessed for larvae feeding on birch^56^. Nevertheless, in the present work we found no significant inter-population difference in the survival rate and hatching success of insects feeding on larch, despite the fact that insects from the Altai population are known to utilize larch as host as frequently as birch.

The survival rate of insects from both populations feeding on larch did not differ significantly from that of those feeding on birch. Furthermore, no difference in survival on pine was observed among insects from the Altai population, known to feed on conifers such as larch, a trend that persisted into the second generation. It has been previously demonstrated that the use of pine (*Pinus rigida*) as a secondary host plant has a positive effect on the survival, pupal mass, and fecundity of the spongy moth^57^. Our own findings indicate that feeding on non-preferred host plants does not result in long-term negative consequences for insect survival. The number of eggs laid decreased in both populations when fed on pine compared to both birch and larch, but remained unchanged on larch compared to birch, confirming a higher degree of adaptation to larch as a transitional host plant. Furthermore, insects of the Altai population had greater pupal mass and, therefore, produced a greater number of eggs in the first generation overall. This phenomenon may be interpreted as an adaptive strategy to increase the chances that at least some offspring will survive under suboptimal conditions. Notably, the Altai population exhibited reduced fecundity on birch in the second generation, while maintaining high reproductive output on larch, their native coniferous host. In contrast, the Novosibirsk population performed best on birch, consistent with its origin in a birch-dominated lowland habitat. Under laboratory uniform conditions, the intrinsic adaptive divergence between populations became evident, particularly the cost of maladaptation experienced by the Altai population. However, variations in egg count across different host plants did not affect hatching success.

Our observation that feeding on pine significantly reduced pupal mass and prolonged larval development in both the Novosibirsk and Altai populations aligns closely with studies on Northern American conifers, where similarly impaired growth and extended development times were observed. Pine foliage consistently supported lower survival and reduced pupal mass compared to deciduous hosts such as *Quercus velutina*, a pattern we also observed with birch as the preferable host^28^. In contrast to pine, larch imposed milder effects particularly in the Altai population, which showed no significant reduction in female pupal mass. The population-specific response we observed suggests local adaptation in the Altai population to larch, a tree species common in its native mountainous habitat. Regarding fecundity, a strong positive correlation between female pupal mass and egg production was established^58^. While we observed reduced pupal mass in Novosibirsk females on larch, egg numbers did not differ significantly from those on birch. This apparent decoupling may reflect short-term compensatory mechanisms.

The present study investigated the physiological adaptations in the digestive system associated with shifts in consumed host plant. The study was conducted with a focus on sex differences, given the mounting evidence of sex-specific physiological responses that extend beyond the reproductive system^59–63^. It is well established that nutrition influences both the overall protease activity and the specific proteases^64^. Plants have evolved a defence mechanism against herbivorous insects in the form of protease inhibitors^65^, to which insects can adapt by overproducing proteases^66–68^ or synthesizing protease isoforms that are insensitive to these inhibitors^69,70^. Although feeding on pine is not typical for either studied populations, and larch is not common for the Novosibirsk population, both populations were able to survive and develop, indicating sufficient adaptation to the protease inhibitors^71–73^ and other secondary metabolites such as tannins^74^ that may modulate proteolytic activity present in these plants.

Feeding on non-preferred host plants has been shown to result in a reduction of larval weight gain, attributable to diminished digestibility and nutrient availability^75,76^. Consequently, an increased production of proteases may act as a compensatory mechanism^33^. In the present study, we observed a decline in female mass gain in the Novosibirsk population individuals fed larch or pine instead of birch; however, changes in protease activity were evident in both populations and were solely influenced by the host plant. The reduced bioavailability of dietary proteins may result from multiple interacting factors, including the protein content of the host plant, the formation of complexes between food proteins and secondary metabolites (e.g., tannins, phenolics), and direct interactions between these allelochemicals and midgut proteolytic enzymes. Additionally, variations in food quality can influence feeding behavior, leading to differences in food intake, while secondary compounds may also alter gut pH or induce detoxification responses that indirectly affect digestive efficiency. Together, these mechanisms can significantly impair protein digestion and nutrient assimilation in herbivorous insects. However, even feeding on evolutionarily unfamiliar plant species containing such metabolites does not always lead to mortality in *Lymantria dispar*^77^, underlining the high degree of polyphagy and adaptability of the species.

The necessity of detoxifying plant secondary metabolites when feeding on novel host plants leads to alterations in esterase activity. Enhanced expression of esterases may contribute to more effective detoxification of secondary metabolites^78^, although esterase overexpression is not always energetically favorable. However, the presence of larch in the natural diet of the Altai population may explain the higher esterase expression observed during feeding on pine, whereas for the Novosibirsk population, this response appears to be energetically costly or detoxification may rely on alternative mechanisms. In the present study, increases in esterase activity were exclusively detected in male subjects. Sex-specific responses to xenobiotics have been documented in various insect species^79,80^. Conifer-derived xenobiotics may modulate esterase activity in insects ^81,82^. For instance, certain xenobiotics have been observed to increase esterase gene expression in male *Drosophila*, while decreasing it in females^83^. The elevated esterase activity observed in the Altai population may facilitate more efficient detoxification of secondary metabolites present in pine, such as sesquiterpenes, α-pinene, limonene, and others^84^, although further investigation is required to confirm this hypothesis.

Oxidative stress, defined as an imbalance favoring oxidants over antioxidants, disrupts redox signaling and is marked by oxidative damage to proteins, lipids, and cellular functions, often assessed through malondialdehyde (MDA), a product of lipid peroxidation^85^. MDA, a known marker of oxidative stress formed during the oxidation of polyunsaturated fatty acids in cell membranes, is an indicator of uncontrolled lipid peroxidation, which in turn compromises membrane integrity^86^ by forming mutagenic adducts with proteins and DNA^87^, as evidenced by its accumulation in insects exposed to toxins and adverse conditions^88–91^ or plant allelochemicals^92,93^. The presence of antioxidants in the diet has been demonstrated to reduce lipid peroxidation^94^. Compounds like betulin, monoterpenes, and polyphenols from plants exhibit protective antioxidant activity^84,95–98^. The elevated MDA levels observed in larvae feeding on larch may result from oxidative damage to midgut epithelial cell membranes, likely induced by pro-oxidant secondary metabolites present in larch needles (e.g., diterpenoids)^24^. Furthermore, it has been demonstrated that plant defense compounds have the capacity to disrupt cell membranes and promote inflammation, thereby further exacerbating lipid peroxidation^99^.

## Conclusions

The findings of the present study show that, irrespective of population origin, the spongy moth (*Lymantria dispar*) has the capacity to feed and produce viable offspring on non-preferred coniferous species, such as pine. This capacity is saved for at least two generations of herbivores. This finding is consistent with previous studies showing that the adaptability of the spongy moth to consume North American species of coniferous hosts depends more on population-specific traits than on subspecies classification^28^. However, the presence of “transitional” or conditionally favorable coniferous species, such as larch, in the insect’s diet enhances the ability of populations to establish themselves on new, previously non-preferred coniferous hosts. This, in combination with higher genetic diversity of Altai population (Martemyanov et al., unpublished data) as compared with populations from Siberian steppes^16^, enables the Altai population to make such transitions more successfully.

However, a number of detrimental effects are associated with the adoption of non-preferred host plants, thereby creating uncertainty regarding the possibility that stable populations capable of forming outbreak-level infestations will become established in northern regions. Nevertheless, the development of novel approaches for the control of spongy moth populations appears to be a promising research direction, given the species’ potential to expand into new geographic areas.

## Supplementary Information

Below is the link to the electronic supplementary material.

Supplementary Material 1

## Data Availability

The data used and analyzed during the current study are available from the corresponding author upon request.

## References

[1] Houghton, J. T. et al.* Climate change 1995 : The science of climate change: edited by J.T. Houghton, L.G. Meira Filho, B.A. Callander, N. Harris, A. Kattenberg and K. Maskell.* (Cambridge University Press, for the Intergovernmental Panel on Climate Change, 1996).

[2] Houghton, J. T. & I, I. P. on C. C. W. G. *Climate change 2001 :The Scientific Basis.* Edited by J.T. Houghton.. [et al.]. (Cambridge University Press, 2001).

[3] Bale, J. S. et al. Herbivory in global climate change research: direct effects of rising temperature on insect herbivores. *Glob. Change Biol.* **8**, 1–16 (2002).

[4] Johnson, D. M. & Haynes, K. J. Spatiotemporal dynamics of forest insect populations under climate change. *Curr. Opin. Insect Sci.* **56**, 101020 (2023). 36906142 10.1016/j.cois.2023.101020

[5] Schaphoff, S. et al. Terrestrial biosphere carbon storage under alternative climate projections. *Clim. Change* **74**, 97–122 (2006).

[6] Vindstad, O. P. L., Jepsen, J. U., Ek, M., Pepi, A. & Ims, R. A. Can novel pest outbreaks drive ecosystem transitions in northern-boreal birch forest?. *J. Ecol.* **107**, 1141–1153 (2019).

[7] Jepsen, J. U., Hagen, S. B., Ims, R. A. & Yoccoz, N. G. Climate change and outbreaks of the geometrids *Operophtera brumata* and *Epirrita autumnata* in subarctic birch forest: Evidence of a recent outbreak range expansion. *J. Anim. Ecol.* **77**, 257–264 (2008). 18070041 10.1111/j.1365-2656.2007.01339.x

[8] Pureswaran, D. S., Roques, A. & Battisti, A. Forest Insects and Climate Change. *Curr. Fore Rep.* **4**, 35–50 (2018).

[9] Pureswaran, D. S. et al. Climate-induced changes in host tree–insect phenology may drive ecological state-shift in boreal forests. *Ecology* **96**, 1480–1491 (2015).

[10] Weed, A. S., Ayres, M. P. & Hicke, J. A. Consequences of climate change for biotic disturbances in North American forests. *Ecol. Monogr.* **83**, 441–470 (2013).

[11] Chapin, F. S. et al. Role of land-surface changes in arctic summer warming. *Science* **310**, 657–660 (2005). 16179434 10.1126/science.1117368

[12] Lenton, T. M. et al. Tipping elements in the Earth’s climate system. *Proc. Natl. Acad. Sci.* **105**, 1786–1793 (2008). 18258748 10.1073/pnas.0705414105 PMC2538841

[13] Scheffer, M., Hirota, M., Holmgren, M., Van Nes, E. H. & Chapin, F. S. Thresholds for boreal biome transitions. *Proc. Natl. Acad. Sci.* **109**, 21384–21389 (2012). 23236159 10.1073/pnas.1219844110 PMC3535627

[14] Kallio, P. & Lehtonen, J. Birch forest damage caused by Oporinia autumnata (Bkh.) in 1965–66 in Utsjoki, N Finland. *Rep. Kevo Subarctic Res.* 55–69 (1973).

[15] Pogue, M. *A Review of Selected Species of Lymantria Hübner (1819) (Lepidoptera: Noctuidae: Lymantriinae) from Subtropical and Temperate Regions of Asia, Including the Descriptions of Three New Species, Some Potentially Invasive to North America* (U.S. Department of Agriculture, 2007).

[16] Martemyanov, V. et al. Genetic evidence of broad spreading of Lymantria dispar in the West Siberian Plain. *PLoS ONE* **14**, e0220954 (2019). 31430316 10.1371/journal.pone.0220954 PMC6701763

[17] Sharov, A. A., Leonard, D., Liebhold, A. M., Roberts, E. A. & Dickerson, W. “Slow the spread”: A national program to contain the gypsy moth. *J. For.* **100**, 30–36 (2002).

[18] Yasyukevich, V. V., Titkina, C. N., Davidovich, E. A. & Yasyukevich, N. V. Changes in range boundaries of the gypsy moth and the nun moth (*Lymantria**dispar*, L. *monacha*, Lymantriidae, Lepidoptera) due to the global warming: a model approach. *Entmol. Rev.* **95**, 1144–1148 (2015).

[19] Iliynykh, A. V. & Krivets (Komarova), S. A. Results of pheromone monitoring of the gypsy moth Lymantria dispar (L.) (Lepidoptera: Lymantriidae) in southeastern Western Siberia. *Proceedings of the Saint-Petersburg Forestry Academy* 45–53 (2011).

[20] Ponomarev, V. I. et al. Phenological features of the spongy moth, *Lymantria**dispar* (L.) (Lepidoptera: Erebidae), in the Northernmost portions of Its Eurasian range. *Insects* **14**, 276 (2023). 36975961 10.3390/insects14030276 PMC10057557

[21] Lovett, G. M., Canham, C. D., Arthur, M. A., Weathers, K. C. & Fitzhugh, R. D. Forest ecosystem responses to exotic pests and pathogens in Eastern North America. *Bioscience* **56**, 395–405 (2006).

[22] Evans, P. & Brown, C. D. The boreal–temperate forest ecotone response to climate change. *Environ. Rev.* **25**, 423–431 (2017).

[23] Stark, S., Julkunen-Tiitto, R., Holappa, E., Mikkola, K. & Nikula, A. Concentrations of foliar quercetin in natural populations of white birch (*Betula**pubescens*) increase with latitude. *J. Chem. Ecol.* **34**, 1382–1391 (2008). 18946705 10.1007/s10886-008-9554-8

[24] Subbotina, A., Chernyak, E., Soukhovolsky, V., Morozov, S. & Martemyanov, V. Latitudinal variation in constitutive chemical defense compounds in two host plants of *Lymantria**dispar* (Lymantriidae): *Betula**pendula* (Betulaceae) and *Larix**sibirica* (Pinaceae). *For. Ecol. Manag.* **590**, 122811 (2025).

[25] Davidson, C. B., Gottschalk, K. W. & Johnson, J. E. Tree mortality following defoliation by the European gypsy moth (*Lymantria**dispar* L.) in the United States: A review. *For. Sci.* **45**, 74–84 (1999).

[26] Soukhovolsky, V., Kovalev, A., Tarasova, O. & Martemyanov, V. Regulatory characteristics of population density dynamics of forest insects and possible reasons for the observed narrow range of such characteristics. *Chaos Solit. Fractals* **191**, 115949 (2025).

[27] Soukhovolsky, V. G., Ponomarev, V. I., Sokolov, G. I., Tarasova, O. V. & Krasnoperova, P. A. Gypsy moth *Lymantria**dispar* L. in the southern Urals: Patterns in population dynamics and modeling. *Biol. Bull. Rev.* **6**, 57–69 (2015). 26201216

[28] Keena, M. A. & Richards, J. Y. Comparison of survival and development of gypsy moth *Lymantria**dispar* L. (Lepidoptera: Erebidae) populations from different geographic areas on North American Conifers. *Insects* **11**, 260 (2020). 32344583 10.3390/insects11040260 PMC7240718

[29] Miller, J. C. & Hanson, P. E. Laboratoru studies on development of gypsy moth, *Lymantria**dispar* (L.) (Lepidoptera: Lymantriidae), larvae on foliage of gymnosperms. *Can. Entomol.* **121**, 425–429 (1989).

[30] Clavijo McCormick, A., Arrigo, L., Eggenberger, H., Mescher, M. C. & De Moraes, C. M. Divergent behavioural responses of gypsy moth (*Lymantria**dispar*) caterpillars from three different subspecies to potential host trees. *Sci. Rep.* **9**, 8953 (2019). 31222054 10.1038/s41598-019-45201-3 PMC6586621

[31] Ponomarev, V. I., Klobukov, G. I., Napalkova, V. V., Tyurin, M. V. & Martemyanov, V. V. Influence of biotic and abiotic factors on the duration of development of the spongy moth *Lymantria**dispar* (L.) (Lepidoptera: Erebidae) in the West Siberian population of different latitudinal origin. *Contemp. Probl. Ecol.* **16**, 166–172 (2023).

[32] Lazarević, J. & Janković-Tomanić, M. Dietary and phylogenetic correlates of digestive trypsin activity in insect pests. *Entomol. Exp. Appl.* **157**, 123–151 (2015).

[33] Pilon, A. M., Oliveira, M. G. A. & Guedes, R. N. C. Protein digestibility, protease activity, and post-embryonic development of the velvetbean caterpillar (Anticarsia gemmatalis) exposed to the trypsin-inhibitor benzamidine. *Pestic. Biochem. Physiol.* **86**, 23–29 (2006).

[34] Napoleão, T. H. et al. Insect midgut structures and molecules as targets of plant-derived protease inhibitors and lectins. *Pest Manag. Sci.* **75**, 1212–1222 (2019). 30306668 10.1002/ps.5233

[35] Lazarević, J., Milanović, S., Šešlija Jovanović, D. & Janković-Tomanić, M. Temperature- and diet-induced plasticity of growth and digestive enzymes activity in spongy moth larvae. *Biomolecules* **13**, 821 (2023). 37238690 10.3390/biom13050821 PMC10216847

[36] Ivashov, A. V., Simchuk, A. P. & Medvedkov, D. A. Possible role of inhibitors of trypsin-like proteases in the resistance of oaks to damage by oak leafroller *Tortrix**viridana* L. and gypsy moth *Lymantria**dispar* L.. *Ecol. Entomol.* **26**, 664–668 (2001).

[37] Schopf, R. The effect of secondary needle compounds on the development of phytophagous insects. *For. Ecol. Manag.* **15**, 55–64 (1986).

[38] Krishnan, N. & Kodrík, D. Antioxidant enzymes in *Spodoptera**littoralis* (Boisduval): Are they enhanced to protect gut tissues during oxidative stress?. *J. Insect Physiol.* **52**, 11–20 (2006). 16242709 10.1016/j.jinsphys.2005.08.009

[39] Picq, S. et al. Range-wide population genomics of the spongy moth, *Lymantria dispar* (Erebidae): Implications for biosurveillance, subspecies classification and phylogeography of a destructive moth. *Evol. Appl.* **16**, 638–656 (2023). 36969137 10.1111/eva.13522 PMC10033852

[40] Ananko, G. G., Kolosov, A. V. & Martemyanov, V. V. Rock microhabitats provide suitable thermal conditions for overwintering insects: A case study of the spongy moth (*Lymantria**dispar* L.) population in the Altai mountains. *Insects* **13**, 712 (2022). 36005337 10.3390/insects13080712 PMC9409708

[41] Ananko, G. G. & Kolosov, A. V. Asian gypsy moth (*Lymantria**dispar* L.) populations: Tolerance of eggs to extreme winter temperatures. *J. Therm. Biol.* **102**, 103123 (2021). 34863486 10.1016/j.jtherbio.2021.103123

[42] Safronova, I. & Yurkovsksya, T. The latitudinal distribution of vegetation cover in Siberia. *BIO Web Conf.* **16**, 00047 (2019).

[43] Schulte, L. et al. Larix species range dynamics in Siberia since the Last Glacial captured from sedimentary ancient DNA. *Commun. Biol.* **5**, 570 (2022). 35681049 10.1038/s42003-022-03455-0 PMC9184489

[44] Wang, C. Z. & Qin, J. D. Partial characterization of protease activity in the midgut of *Helicoverpa**armigera* larvae. *Acta Entomol. Sin*10.16380/j.kcxb.1996.01.002 (1996).

[45] Prabhakaran, S. K. & Kamble, S. T. Biochemical characterization and purification of esterases from three strains of German cockroach, *Blattella**germanica* (Dictyoptera: Blattellidae). *Arch. Insect Biochem. Physiol.* **31**, 73–86 (1996).

[46] Senthilkumar, M., Amaresan, N. & Sankaranarayanan, A. Estimation of Malondialdehyde (MDA) by Thiobarbituric Acid (TBA) Assay. In *Plant-Microbe Interactions: Laboratory Techniques* (eds. Senthilkumar, M., Amaresan, N. & Sankaranarayanan, A.) 103–105 (Springer US, New York, NY, 2021). 10.1007/978-1-0716-1080-0_25.

[47] Hammer, Ø. & Harper, D. A. T. Past: paleontological statistics software package for educaton and data anlysis. *Palaeontol. Electron.* **4**, 1 (2001).

[48] Brooks, M. E. et al. glmmTMB balances speed and flexibility among packages for zero-inflated generalized linear mixed modeling. *R J.* **9**, 378–400 (2017).

[49] The Comprehensive R Archive Network. https://cran.r-project.org/.

[50] Kostić, I. et al. Potential of essential oils from anise, dill and fennel seeds for the Gypsy Moth control. *Plants* **10**, 2194 (2021). 34686003 10.3390/plants10102194 PMC8538750

[51] Sohail, M., Aqueel, M. A., Dai, P. & Ellis, J. D. The larvicidal and adulticidal effects of selected plant essential oil constituents on greater wax moths. *J. Econ. Entomol.* **114**, 397–402 (2021). 33558901 10.1093/jee/toaa249

[52] Powell, J. S. & Raffa, K. F. Effects of selected *Larix**laricina* Terpenoids on *Lymantria**dispar* (Lepidoptera: Lymantriidae) development and behavior. *Environ. Entomol.* **28**, 148–154 (1999).

[53] Mrdaković, M. et al. Adaptive phenotypic plasticity of gypsy moth digestive enzymes. *Cent. Eur. J. Biol.* **9**, 309–319 (2014).

[54] Lazarević, J., Perić Mataruga, V. & Tucić, N. Pre-adult development and longevity in natural populations of *Lymantria**dispar* (Lepidoptera: Lymantriidae). *Eur. J. Entomol.* **104**, 211–216 (2007).

[55] Baranchikov, Yu. N. *Energy Expenditure of Gypsy Silkworm (Lymantria dispar L.) Larvae During Change of a Forage Plant*. In (1981).

[56] Soukhovolsky, V. et al. Economics of a feeding budget: a case of diversity of host plants for *Lymantria**dispar* L. (Lepidoptera) feeding on leaves and needles. *Diversity* **15**, 102 (2023).

[57] Rossiter, M. Use of a secondary host by non-outbreak populations of the gypsy moth. *Ecology* **68**, 857–868 (1987).

[58] Miller, W. E. Extrinsic effects on fecundity-maternal weight relations in capital-breeding Lepidoptera. *J. Lepid. Soc.* **59**, 143–160 (2005).

[59] Belousova, I., Pavlushin, S., Subbotina, A., Rudneva, N. & Martemyanov, V. Sex specificity in innate immunity of insect larvae. *J. Insect Sci.* **21**, 15 (2021). 34865031 10.1093/jisesa/ieab097 PMC8644026

[60] Fotouhi, K. et al. Sex-specific susceptibility of carob moth, Ectomyelois ceratoniae (zeller) (Lepidoptera: Pyralidae), to *Ferula**assa-foetida* L. (apiaceae) essential oil under controlled laboratory conditions. *Biocatal. Agric. Biotechnol.* **56**, 103057 (2024).

[61] Joop, G., Mitschke, A., Rolff, J. & Siva-Jothy, M. T. Immune function and parasite resistance in male and polymorphic female Coenagrion puella. *BMC Evol. Biol.* **6**, 19 (2006). 16522202 10.1186/1471-2148-6-19 PMC1431586

[62] Kurtz, J., Wiesner, A., Götz, P. & Sauer, K. P. Gender differences and individual variation in the immune system of the scorpionfly *Panorpa**vulgaris* (Insecta: Mecoptera). *Dev Comp Immunol* **24**, 1–12 (2000). 10689094 10.1016/s0145-305x(99)00057-9

[63] Nunn, C. L., Lindenfors, P., Pursall, E. R. & Rolff, J. On sexual dimorphism in immune function. *Philos. Trans. R. Soc. Lond. B Biol. Sci.* **364**, 61–69 (2009). 18926977 10.1098/rstb.2008.0148 PMC2666693

[64] Rocha, F. A. D. et al. Effect of natural and artificial diets on protease activity in the midgut of *Spodoptera**cosmioides* and *Spodoptera**eridania* (Lepidoptera: Noctuidae) Larvae. *Fla. Entomol.* **103**, 452–457 (2021).

[65] Divekar, P. A. et al. Protease inhibitors: An induced plant defense mechanism against herbivores. *J. Plant Growth Regul.* **42**, 6057–6073 (2023).

[66] Bown, D. P., Wilkinson, H. S. & Gatehouse, J. A. Regulation of expression of genes encoding digestive proteases in the gut of a polyphagous lepidopteran larva in response to dietary protease inhibitors. *Physiol. Entomol.* **29**, 278–290 (2004).

[67] Meriño-Cabrera, Y. et al. Biochemical response between insects and plants: an investigation of enzyme activity in the digestive system of *Leucoptera coffeella* ( *Lepidoptera* : *Lyonetiidae* ) and leaves of *Coffea arabica* ( *Rubiaceae* ) after herbivory. *Ann. Appl. Biol.* **172**, 236–243 (2018).

[68] Zhao, A., Li, Y., Leng, C., Wang, P. & Li, Y. Inhibitory effect of protease inhibitors on larval midgut protease activities and the performance of *Plutella**xylostella* (Lepidoptera: Plutellidae). *Front. Physiol.* **9**, 1963 (2019). 30697169 10.3389/fphys.2018.01963 PMC6340996

[69] Jongsma, M. A., Bakker, P. L., Peters, J., Bosch, D. & Stiekema, W. J. Adaptation of Spodoptera exigua larvae to plant proteinase inhibitors by induction of gut proteinase activity insensitive to inhibition. *Proc. Natl. Acad. Sci. U.S.A.* **92**, 8041 (1995). 7644535 10.1073/pnas.92.17.8041 PMC41282

[70] Jongsma, M. A. & Bolter, C. The adaptation of insects to plant protease inhibitors. *J. Insect Physiol.* **43**, 885–895 (1997). 12770458 10.1016/s0022-1910(97)00040-1

[71] Meng, Z., Tong, L., Gao, L., Yan, S. & Lu, Y. Activities of some defense proteins associated with age or plant family in larch needles of *Larix**olgensis* and L. *kaempferi* × L. *gmelinii*. *J. For. Res.* **28**, 63–69 (2017).

[72] Qi, W. Activities of proteinase inhibitors in *Larix**gmelinii* seedlings under the stresses of cutting needles and herbivore feeding. *Acta Entomol. Sin.* **51**, 798–803 (2008).

[73] Zhang Jian, Z. J., Yan ShanChun, Y. S. & Wang Qi, W. Q. Influence of the different cone grades on activities of protective enzymes and protease inhibitors in needles of Larix spp. *Sci. Silvae Sin.* **45**, 96–100 (2009).

[74] Barbehenn, R. V. & Constabel, P. C. Tannins in plant-herbivore interactions. *Phytochemistry* **72**, 1551–1565 (2011). 21354580 10.1016/j.phytochem.2011.01.040

[75] Soo Hoo, C. F. & Fraenkel, G. The consumption, digestion, and utilization of food plants by a polyphagous insect, *Prodenia eridania* (Cramer). *J. Insect Phys.* **12**, 711–730 (1966).

[76] Stoyenoff, J. L., Witter, J. A., Montgomery, M. E. & Chilcote, C. A. Effects of host switching on gypsy moth (*Lymantria**dispar* (L.)) under field conditions. *Oecologia* **97**, 143–157 (1994). 28313923 10.1007/BF00323144

[77] Matsuki, M., Kay, N., Serin, J. & Scott, J. K. Variation in the ability of larvae of phytophagous insects to develop on evolutionarily unfamiliar plants: a study with gypsy moth *Lymantria**dispar* and Eucalyptus. *Agric. For. Entomol.* **13**, 1–13 (2011).

[78] War, A. R. et al. Plant Defense and Insect Adaptation with Reference to Secondary Metabolites. in *Co-Evolution of Secondary Metabolites* (eds. Merillon, J.-M. & Ramawat, K. G.) 1–28 (Springer International Publishing, Cham, 2019). 10.1007/978-3-319-76887-8_60-1.

[79] Tang, R. et al. Transcriptomics and metagenomics of common cutworm (Spodoptera litura) and fall armyworm (Spodoptera frugiperda) demonstrate differences in detoxification and development. *BMC Genomics* **23**, 388 (2022). 35596140 10.1186/s12864-022-08613-6 PMC9123734

[80] Thornton, B. J. *Sex-dependent changes in activity of detoxification enzymes, insecticide susceptibility, and alterations in protein expression induced by atrazine in Drosophila melanogaster* 1–141 (ETD collection for University of Nebraska-Lincoln, 2009).

[81] Liu, B. & Chen, H. Disruption of carboxylesterase DaEST3 reduces tolerance to host allelochemicals in Dendroctonus armandi. *Arthropod-Plant Interact.* **17**, 673–685 (2023).

[82] Wang, Z. et al. Conifer flavonoid compounds inhibit detoxification enzymes and synergize insecticides. *Pestic. Biochem. Physiol.* **127**, 1–7 (2016). 26821651 10.1016/j.pestbp.2015.09.003

[83] Le Goff, G. et al. Xenobiotic response in Drosophila melanogaster: Sex dependence of P450 and GST gene induction. *Insect Biochem. Mol. Biol.* **36**, 674–682 (2006). 16876710 10.1016/j.ibmb.2006.05.009

[84] Rogachev, A. D. & Salakhutdinov, N. F. Chemical composition of *Pinus sibirica* (Pinaceae). *Chem. Biodivers.* **12**, 1–53 (2015). 25641836 10.1002/cbdv.201300195

[85] Apel, K. & Hirt, H. Reactive oxygen species: metabolism, oxidative stress, and signal transduction. *Annu. Rev. Plant Biol.* **55**, 373–399 (2004). 15377225 10.1146/annurev.arplant.55.031903.141701

[86] Li, J., Kang, R. & Tang, D. Monitoring autophagy-dependent ferroptosis. *Methods Cell Biol* **165**, 163–176 (2021). 34311865 10.1016/bs.mcb.2020.10.012

[87] Marín, R., Abad, C., Rojas, D., Chiarello, D. I. & Alejandro, T.-G. Biomarkers of oxidative stress and reproductive complications. *Adv. Clin. Chem.* **113**, 157–233 (2023). 36858646 10.1016/bs.acc.2022.11.004

[88] Coates, C. J. et al. The insect, Galleria mellonella, is a compatible model for evaluating the toxicology of okadaic acid. *Cell Biol. Toxicol.* **35**, 219–232 (2019). 30426330 10.1007/s10565-018-09448-2 PMC6556153

[89] Mao, C., Lei, G., Horbath, A. & Gan, B. Assessment of lipid peroxidation in irradiated cells. *Methods Cell Biol.* **172**, 37–50 (2022). 36064225 10.1016/bs.mcb.2022.05.003 PMC11881802

[90] Miao, Z. Q. et al. Antioxidant enzymes and heat shock protein genes from Liposcelis bostrychophila are involved in stress defense upon heat shock. *Insects* **11**, 839 (2020). 33261171 10.3390/insects11120839 PMC7759835

[91] Wang M. et al. Dietary oxidized lipids. In *Food Lipids* 349–380 (Academic Press, 2022). 10.1016/B978-0-12-823371-9.00006-X.

[92] Tan, M., Wu, H., Yan, S. & Jiang, D. Evaluating the toxic effects of tannic acid treatment on *Hyphantria**cunea* larvae. *Insects* **13**, 872 (2022). 36292820 10.3390/insects13100872 PMC9604457

[93] Xue, M. et al. Identification and functional analysis of an epsilon class glutathione S-transferase gene associated with α-Pinene adaptation in *Monochamus**alternatus*. *Int. J. Mol. Sci.* **24**, 17376 (2023). 38139205 10.3390/ijms242417376 PMC10743883

[94] Cohen, A. C. & Crittenden, P. Deliberately added and “cryptic” antioxidants in three artificial diets for insects. *J. Econ. Entomol.* **97**, 265–272 (2004). 15154444 10.1603/0022-0493-97.2.265

[95] Kalugina, O. V., Afanasyeva, L. V., Mikhailova, T. A. & Filinova, N. V. Activity of low-molecular weight components of Larix sibirica antioxidant system under exposure to technogenic pollution. *Ecotoxicology* **31**, 1492–1505 (2022). 36445649 10.1007/s10646-022-02607-6

[96] Ostapiuk, A., Kurach, Ł, Strzemski, M., Kurzepa, J. & Hordyjewska, A. Evaluation of antioxidative mechanisms in vitro and triterpenes composition of extracts from silver birch (Betula pendula Roth) and black birch (Betula obscura Kotula) Barks by FT-IR and HPLC-PDA. *Molecules* **26**, 4633 (2021). 34361786 10.3390/molecules26154633 PMC8347892

[97] Popescu (Stegarus), D. I. et al. Comparative Antioxidant and Antimicrobial Activities of Several Conifer Needles and Bark Extracts. *Pharmaceutics* **16**, 52 (2024). 10.3390/pharmaceutics16010052 PMC10821083 38258063

[98] Salehi, B. et al. Therapeutic potential of α- and β-pinene: A miracle gift of nature. *Biomolecules* **9**, 738 (2019). 31739596 10.3390/biom9110738 PMC6920849

[99] Sottero, B., Rossin, D., Poli, G. & Biasi, F. Lipid oxidation products in the pathogenesis of inflammation-related gut diseases. *CMC* **25**, 1311–1326 (2018). 10.2174/0929867324666170619104105 28625152

