# Supplementary material for: Adaptive capacity of Asian populations of Lymantria dispar to non preferred plants during northward expansion
Source: Sci Rep. 2026 Jan 16;16:2756. doi: 10.1038/s41598-025-32504-x (PMC12824199; doi:10.1038/s41598-025-32504-x)
Supplement: Supplementary file 1 — Supplementary Material 1 [file 41598_2025_32504_MOESM1_ESM.docx]

Supplementary table 1. Results of factorial ANOVA for the number of eggs laid by *Lymantria dispar* in the first generation on different host plants.

|  | SS | Degrees of freedom | MS | F | p |
| --- | --- | --- | --- | --- | --- |
| Intercept | 4811568 | 1 | 4811568 | 1307.826 | 0.000000 |
| Host plant | 356769 | 2 | 178384 | 48.486 | 0.000000 |
| Population | 155347 | 1 | 155347 | 42.225 | 0.000000 |
| Host plant x population | 2586 | 2 | 1293 | 0.351 | 0.705253 |
| Error | 198669 | 54 | 3679 |  |  |

Supplementary table 2. Results of factorial ANOVA for the number of eggs laid by *Lymantria dispar* in the second generation on different host plants.

|  | SS | Degrees of freedom | MS | F | p |
| --- | --- | --- | --- | --- | --- |
| Intercept | 3629976 | 1 | 36299976 | 563.8266 | 0.000000 |
| Host plant | 197368 | 2 | 98684 | 15.3281 | 0.000005 |
| Population | 39938 | 1 | 39938 | 6.2034 | 0.015857 |
| Host plant x population | 2008627 | 2 | 104314 | 16.2025 | 0.000003 |
| Error | 347658 | 54 | 6438 |  |  |

Supplementary table 3. Results of factorial ANOVA for the pupal mass of *Lymantria dispar* on different host plants.

|  | SS | Degrees of freedom | MS | F | p |
| --- | --- | --- | --- | --- | --- |
| Intercept | 327165251 | 1 | 327165251 | 14671.28 | 0.000000 |
| Host plant | 18508582 | 2 | 9254291 | 415.00 | 0.000000 |
| Population | 11334109 | 1 | 11334109 | 508.26 | 0.000000 |
| Sex | 42672998 | 1 | 42672998 | 1913.61 | 0.000000 |
| Host plant x population | 595071 | 2 | 297535 | 13.34 | 0.000002 |
| Host plant x sex | 3796434 | 2 | 1898217 | 85.12 | 0.000000 |
| Population x sex | 3425302 | 1 | 3425302 | 153.60 | 0.000000 |
| Host plant x population x sex | 306024 | 2 | 153012 | 6.68 | 0.001132 |
| Error | 13357523 | 599 | 22300 |  |  |

Supplementary table 4. Results of factorial ANOVA for the alkaline proteases activity in the midgut of *Lymantria dispar* on different host plants.

|  | SS | Degrees of freedom | MS | F | p |
| --- | --- | --- | --- | --- | --- |
| Intercept | 720.5600 | 1 | 720.5600 | 569.1012 | 0.000000 |
| Host plant | 111.6064 | 2 | 55.8032 | 44.0736 | 0.000000 |
| Population | 1.1324 | 1 | 1.1324 | 0.8944 | 0.346192 |
| Sex | 1.2709 | 1 | 1.2709 | 1.0038 | 0.318414 |
| Host plant x population | 2.0634 | 2 | 1.0317 | 0.8148 | 0.445144 |
| Host plant x sex | 1.7002 | 2 | 0.8501 | 0.6714 | 0.512886 |
| Population x sex | 0.2016 | 1 | 0.2016 | 0.1592 | 0.690580 |
| Host plant x population x sex | 2.0131 | 2 | 1.0065 | 0.7950 | 0.453963 |
| Error | 151.9364 | 120 | 1.2661 |  |  |
